# Serum γ-Glutamyltransferase Concentration Predicts Endothelial Dysfunction in Naïve Hypertensive Patients

**DOI:** 10.3390/biomedicines8070207

**Published:** 2020-07-11

**Authors:** Maria Perticone, Raffaele Maio, Benedetto Caroleo, Angela Sciacqua, Edoardo Suraci, Simona Gigliotti, Francesco Martino, Francesco Andreozzi, Giorgio Sesti, Francesco Perticone

**Affiliations:** 1 Department of Experimental and Clinical Medicine, Magna Graecia University, 88100 Catanzaro, Italy; 2 Geriatrics Division, University Hospital Mater Domini, 88100 Catanzaro, Italy; raf_maio@yahoo.it (R.M.); benedettocaroleo@libero.it (B.C.); 3 Department of Medical and Surgical Sciences, Magna Graecia University, 88100 Catanzaro, Italy; sciacqua@unicz.it (A.S.); edoardosuraci88@gmail.com (E.S.); simona_gigliotti@yahoo.it (S.G.); andreozzif@unicz.it (F.A.); perticone@unicz.it (F.P.); 4 Department of Pediatrics Gynecology and Obstetrics, Sapienza University of Rome, 00185 Rome, Italy; francesco.martino30@tin.it; 5 Department of Clinical and Molecular Medicine, Sant’ Andrea University Hospital, Sapienza University of Rome, 00185 Rome, Italy; giorgio.sesti@uniroma1.it

**Keywords:** serum γ-glutamyltransferase, endothelial dysfunction, essential hypertension, atherosclerosis, cardiovascular risk factors

## Abstract

Background: Serum gamma-glutamyltransferase (γ-GT) is recognized as a risk factor for cardiovascular diseases (CV). Traditional cardiovascular risk factors mediate endothelial dysfunction. Aim: to evaluate a possible correlation between serum γ-GT and endothelium-dependent vasodilation in naïve hypertensives. Methods: We enrolled 500 hypertensives. Endothelial function was studied by strain-gauge plethysmography. Receiver operating characteristic (ROC) analysis was used to assess the predictive value of γ-GT and to identify the optimal cut-off value of the same variable for endothelial dysfunction. Results: At univariate linear analysis peak percent increase in acetylcholine (ACh)-stimulated vasodilation was inversely related to γ-GT (*r* = −0.587), alanine aminotransferase (ALT) (*r* = −0.559), aspartate aminotransferase (AST) (*r* = −0.464), age (*r* = −0.171), body mass index (BMI) (*r* = −0.152), and fasting glucose (*r* = −101). In the stepwise multivariate regression model, endothelium-dependent vasodilation was significantly related to γ-GT (β = −0.362), ALT (β = −0.297), AST (β = −0.217), estimated glomerular filtration rate (e-GFR) (β = 0.199), gender (β = 0.166), and smoking (β = −0.061). The ROC analysis demonstrated that the accuracy of γ-GT for identifying patients with endothelial dysfunction was 82.1%; the optimal γ-GT cut-off value for discriminating patients with this alteration was 27 UI/L. Conclusions: Serum γ-GT values, within the normal range, are significantly associated with endothelial dysfunction in hypertensives, and may be considered a biomarker of early vascular damage.

## 1. Introduction

Gamma-glutamyltransferase (γ-GT) is a glycoprotein located on the plasma membranes of most cells and organ tissues, especially of hepatocytes. It is involved in the extracellular catabolism of glutathione, recognized as the major thiol antioxidant in humans and other mammals. An increase in serum γ-GT levels >50 IU/mL is seen as a consequence of liver injury or bile ducts blockage. In clinical practice, serum γ-GT levels are routinely measured when hepatic/biliary disease and/or alcohol abuse are suspected [1], and it has recently been recognized as a risk factor for metabolic alterations [2], and chronic renal [3] and cardiovascular diseases (CV) [4,5,6,7]. These effects seem to be mediated by the capacity of γ-GT to increase the production of reactive oxygen species (ROS) in the presence of some transition metal such as iron [4,8]. Thus, on the basis of these findings and of other epidemiological studies, it was suggested to consider γ-GT, within its normal range, as an early and sensitive biomarker of oxidative stress [9]. In fact, subclinical inflammation related to oxidative stress is considered as the main pathogenetic mechanism involved in several cardio-metabolic diseases [10].

It is well established that endothelial dysfunction, an early event in the atherogenic process, is associated with some metabolic and hemodynamic [11,12,13,14] risk factors (i.e., arterial hypertension, obesity, etc.) sharing the same pathogenetic mechanisms, represented by an increased oxidative stress and subclinical inflammation. The activation of these pro-oxidant and pro-inflammatory pathways leads to the activation and progression of atherosclerotic disease by reducing nitric oxide (NO) bioavailability and its protective effect on vascular function [15,16]. In addition, it is important to remark that endothelial dysfunction has been demonstrated to predict the progression of subclinical target organ damage [17,18,19]; on this basis, it is possible to affirm that endothelial dysfunction has a key role in the pathogenetic mechanisms of CV diseases and associated outcomes [14]. Even if the association between essential hypertension and endothelial dysfunction is well established, to our knowledge there are no data testing a possible relationship between serum γ-GT concentrations and endothelial function in this setting of patients. Thus, we designed the present study to evaluate a possible association between serum γ-GT concentrations, within its normal range, and endothelium-dependent vasodilation, evaluated by strain-gauge plethysmography, in a large population of newly diagnosed, never-treated hypertensive patients.

## 2. Experimental Section

For this study we enrolled 500 Caucasian hypertensive outpatients (256 men and 244 women; mean age, 47 ± 11 years) referred to the Hypertension Clinic of the University Hospital of Catanzaro, Italy. Exclusion criteria were secondary forms of hypertension, clinical evidence or previous history of coronary artery disease, valvular heart disease, peripheral artery disease, diabetes mellitus, hypercholesterolemia, liver diseases, impaired renal function (defined as an estimated glomerular filtration rate [e-GFR] <60 mL/min per 1.73 m^2^), coagulopathies, vasculitis and/or Raynaud’s phenomenon, history of alcohol and/or drug abuse, and the use of drugs interfering with liver enzyme concentrations. To enter the study protocol, all subjects had to have serum values of alanine aminotransferase (ALT), aspartate aminotransferase (AST), and γ-GT in the normal range; in particular, we considered 50 IU/mL as the upper normal limit for all these three variables, as established by our laboratory. At the first evaluation, all subjects underwent routine blood tests, assessment of risk factors for atherosclerosis, and evaluation of vascular function through strain-gauge plethysmography. ALT and AST were measured by pyridoxal phosphate activated (liquid reagent) (COBAS Integra 800—Roche Diagnostics GmbH, Mannheim, Germany; normal values 0–50 UI/L), γ-GT was evaluated by standardized against Szasz (COBAS Integra 800—Roche Diagnostics GmbH, Mannheim, Germany; normal values 8–50 UI/L).

High-sensitivity C-reactive protein (hs-CRP) was measured by a high-sensitivity turbidimetric immunoassay (Cardio-Phase hs-CRP, Siemens Healthcare Diagnostics GmbH, Marburg, Germany) in a subgroup of 400 patients representative of the whole study population with regard to the variables listed in Table 1.

The local ethics committee approved the study (approval number 2012.63, 23 October 2012—Comitato Etico Azienda Ospedaliero-Universitaria Mater Domini of Catanzaro, Italy), and all participants gave written informed consent for all procedures. All the study procedures were conducted according to the Declaration of Helsinki.

### 2.1. Vascular Function Evaluation

Vascular function evaluation was made at 09:00 a.m. in a quiet and air-conditioned room (22–24 °C) with the fasting subjects lying supine. Forearm volume was determined by water displacement. A 20-gauge polyethylene catheter (Vasculon 2; Baxter Healthcare, Deerfield, IL, USA), introduced into the brachial artery of the non-dominant arm, was used for evaluation of blood pressure (BP) and for drug administration. Measurement of percent change in forearm volume was obtained by a mercury-filled silastic strain gauge placed on the widest part of the forearm, connected to a plethysmograph (model EC-4; DE Hokanson, Issaquah, WA, USA) that was connected to a chart recorder for detection of forearm blood flow (FBF) measurements. Exclusion of peripheral venous outflow was obtained by inflating to 40 mmHg a cuff placed on the upper arm with a rapid cuff inflator (model E-10; DE Hokanson, Issaquah, WA, USA). FBF was calculated as the slope of the change in forearm volume; the mean of 3 measurements was obtained at each time point.

For the evaluation of endothelial function, we used the protocol initially described by Panza et al. [12] and subsequently used by us [13,14,17,18]. Endothelium-dependent and endothelium-independent vasodilation was assessed by a dose–response curve during intra-arterial infusions of acetylcholine (ACh) (7.5, 15, and 30 μg/mL per minute, each for 5 min) and sodium nitroprusside (SNP) (0.8, 1.6, and 3.2 μg/mL per minute, each for 5 min), respectively. Prior to the administration, ACh (Sigma, Milan, Italy) was diluted with saline and SNP (Malesci, Florence, Italy) in 5% glucose solution, and protected from light with aluminum foil.

### 2.2. Statistical Analysis

Data are reported as mean ± SD or as percent frequency; we used *t*-test or the χ^2^ test, as appropriate, for comparisons between groups. Relationships between paired parameters were tested by correlation coefficient of Pearson. Multivariate models (linear or logistic regression) were constructed using, as independent covariates, several traditional CV risk factors—age, gender, body mass index, glucose, LDL and HDL cholesterol, triglyceride, BP, smoking, and e-GFR—to test the independent relationship between γ-GT and the response to ACh. In an additional analysis, we tested the potential confounding effect of hs-CRP in a subgroup of 400 patients.

In the logistic regression analysis, endothelial dysfunction, as dichotomic variable, was expressed as a maximal response to ACh < 400% as previously reported [17,18]. 

In multiple linear regression models, data were expressed as standardized regression coefficient (beta) and *p* value. In multiple logistic regression analyses, data were expressed as odds ratio (OR), 95% confidence interval (CI), and *p* value. 

Receiver operating characteristic (ROC) analysis was used to assess the predictive value of γ-GT (area under the curve) and to identify the optimal cut-off value of the same variable for endothelial dysfunction, i.e., the value which maximizes the difference between true positive and false positive rates of endothelial dysfunction. 

To assess the internal consistency of study results, a sensitivity analysis was performed by randomly dividing the whole study population into two equally sized subgroups.

All calculations were made with a standard statistical package (SPSS for Windows version 20.0; SPSS, Inc., Chicago, IL, USA).

## 3. Results

Baseline demographic, clinical, and hemodynamic characteristics of the whole study population and of the subgroup of 400 patients, with hs-CRP dosage, are summarized in Table 1.

### 3.1. Correlational Analysis

The results of univariate linear analysis between ACh-stimulated vasodilation and different covariates in the study population are reported in Table 2. An inverse relationship was found between peak percent increase in ACh-stimulated vasodilation and the following: γ-GT (−0.587), accounting for 34.4% of its variation; ALT (*r* = −0.559), accounting for 31.2% of its variation; AST (*r* = –0.464), accounting for 21.5% of its variation; age (*r* = −0.171), accounting for 2.9% of its variation; BMI (*r* = −0.152), accounting for 2.3% of its variation; and fasting glucose (*r* = −101), accounting for 1% of its variation. On the contrary, a direct relationship was observed with the following covariates: e-GFR (*r* = 0.257), accounting for 6.6% of its variation and HDL cholesterol (*r* = 0.108), accounting for 1.2% of its variation. No significant relationships were detected between ACh-stimulated FBF and systolic and diastolic BP, triglyceride, and LDL cholesterol.

### 3.2. Multivariate Analysis

To evaluate the independent predictors of ACh-stimulated maximal FBF, covariates reaching statistical significance, with the addition of smoking and gender as dichotomic variables, were inserted into a stepwise multivariate regression model (Table 3). Results of this analysis demonstrated that the variables significantly associated with endothelium-dependent vasodilation were γ-GT (β = −0.362; *p* = 0.0001), ALT (β = −0.297; *p* = 0.0001), AST (β = −0.217; *p* = 0.0001), e-GFR (β = 0.199; *p* = 0.0001), gender (β = 0.166; *p* = 0.0001), and smoking (β = −0.061; *p* = 0.044). γ-GT and ALT account for 35.4% and 12.3% of the FBF variation, respectively, while other covariates retained in the final model explain another 9.8% of its variation. Overall, the final model accounts for 56.5% of FBF variation.

In the additional analysis including serum hs-CRP, conducted in a subgroup of 400 patients, the association between serum γ-GT and the peak percent of increase of ACh-stimulated FBF did not change (β = −0.362; *p* = 0.0001). 

Finally, we performed multiple logistic regression analyses to estimate the odds of endothelial dysfunction, adopting the cut-off value of 400% of increase in FBF as the dependent variable, associated with serum γ-GT levels (Table 4). The probability of endothelial dysfunction was significantly increased by γ-GT (OR = 1.927 for 10 UI/L), ALT (OR = 2.175 for 10 UI/L), AST (OR = 1.973 for 10 UI/L), and gender (OR = 2.695 male vs. female); on the contrary, the risk of endothelial function impairment was significantly reduced by the preservation of e-GFR (OR = 0.699 for 10 mL/min/1.73 m^2^). The additional analysis, including serum hs-CRP in the same model, demonstrated that the link between serum γ-GT and endothelial dysfunction did not change (OR = 1.953; 95% CI = 1.527–2.499; *p* = 0.0001).

**Figure 1:**
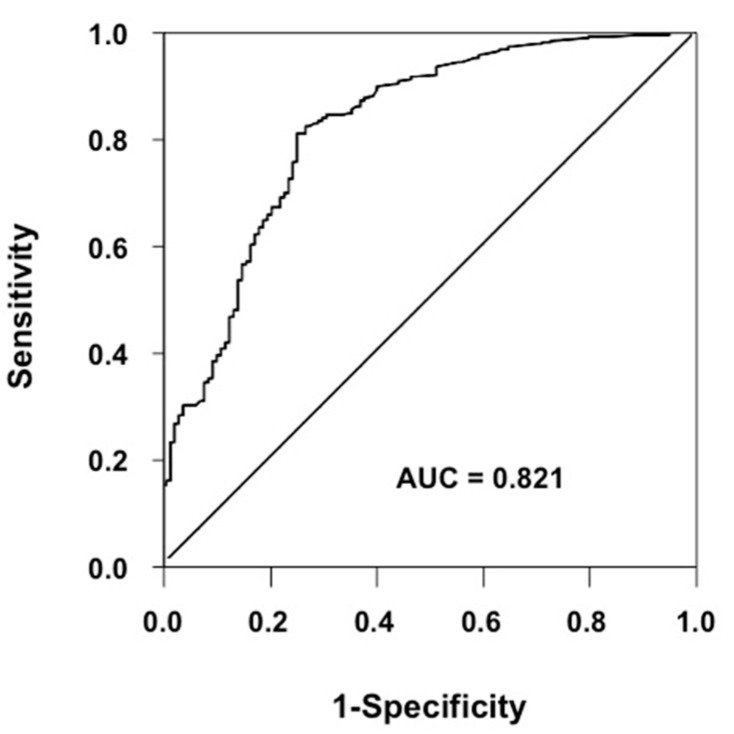
Receiver operating characteristic (ROC) curve for the accuracy of γ-GT for identifying patients with endothelial dysfunction. The accuracy of γ-GT for identifying patients with endothelial dysfunction is 82.1% (AUC = 0.821, *p* < 0.001).

The ROC analysis demonstrated that the accuracy of γ-GT for identifying patients with endothelial dysfunction was 82.1% (AUC = 0.821, *p* < 0.001) (Figure 1) and that the optimal γ-GT cut-off value for discriminating patients with this alteration from those without was 27 UI/L, a threshold providing a 81% sensitivity and a 74% specificity. 

### 3.3. Cross-Validation

To assess the robustness of study results, we performed a sensitivity analysis by randomly dividing the whole study population into two equally sized subgroups. This additional analysis showed that the strength of the relationships between the key risk factors (namely, γ-GT, ALT, AST, gender, and e-GFR) with the outcome variable (endothelial dysfunction) we found in the whole study population (see Table 4) was of similar magnitude to that found in subgroup A (see Table 5) and subgroup B (see Table 6) of the sensitivity analysis. The AUC in the two subgroups (Subgroup A: 76.9%; Subgroup B: 82.1%) provided by the above-mentioned risk factors was almost identical to that found in the whole study population (82.1%), indicating an adequate internal consistency of study results.

## 4. Discussion

To our knowledge, findings obtained in this study demonstrate, for the first time, the association between serum γ-GT within the normal range and endothelial dysfunction, evaluated by strain-gauge plethysmography, in a very large and well-characterized population of never-treated hypertensive patients. Particularly, the risk of endothelial dysfunction increases by 93% for each 10 IU/L elevation of this enzyme (Table 4). 

It is well established that the primary role of γ-GT is to contribute to the maintenance of intracellular homeostasis of glutathione (GSH), one of the major intracellular antioxidant components [20], even if some experimental findings demonstrated that, in the presence of iron or other transition metals, it might also be involved in the generation of ROS [9]. Thus, on the basis of this evidence, γ-GT emerged over time as an early and sensitive enzyme related to oxidative stress. In fact, its circulating levels, within normal range, resulted in the increase of F2-isoprostanes, fibrinogen, and CRP, all markers of systemic inflammation [9]. Interestingly, this association was observed independently of the presence of metabolic alterations, typically related to γ-GT elevation [5,21]. Consistent with these findings, we also observed a significant relationship between γ-GT and hs-CRP, confirming previously published data suggesting that elevation of γ-GT is involved in the subclinical inflammatory response and oxidative stress [22], both conditions associated with endothelial dysfunction [13,23]. 

Another important finding of this study is that ALT levels, within the normal range, are also significantly associated with endothelial function. Particularly, the increase of 10 IU/L of ALT almost doubles the risk of endothelial dysfunction, as reported in Table 4. Although a significant relationship between γ-GT values within the normal range and the incidence of chronic elevation of ALT was previously reported [24], no data are available to demonstrate a possible pathogenetic role of ALT in the activation of an oxidative stress process. Although γ-GT can be considered as an early biomarker of systemic and hepatic oxidative stress, ALT elevation might reflect possible inflammatory liver damage as a consequence of this increased oxidative stress. In accordance with this, we previously reported that hypertensive patients with both metabolic syndrome and non-alcoholic fatty liver disease (NAFLD) had a reduced endothelium-dependent vasodilation in comparison with hypertensives with metabolic syndrome without NAFLD [25]. Serum ALT values were significantly higher in hypertensives with NAFLD than in those without (42.0 + 10.8 vs. 24.6 + 5.2 UI/L), leading to the hypothesis that NAFLD—and the associated elevation of ALT levels—could be considered as an early biomarker of endothelial dysfunction. 

## 5. Conclusions

The results obtained in the present study showed a strong and inverse relationship between γ-GT and endothelium-dependent vasodilation; in particular, the novelty of this paper is the identification of a cut-off value of γ-GT (27 IU/L) to discriminate between patients with and without (γ-GT> and <27 IU/L, respectively) endothelial dysfunction. In addition, present data confirm previously published literature demonstrating the strong associations between serum γ-GT and many CV risk factors and/or events; in this context endothelial dysfunction could be considered as an established consequence of increased oxidative stress. Furthermore, it is important to remark that strain-gauge plethysmography represents the gold-standard technique for endothelial function testing, thus conferring robustness to the results obtained in a wide population. Thus, serum γ-GT may be considered an additional biomarker of early vascular damage; its usefulness is also supported by its wide availability and low cost.

## Figures and Tables

**Table 1:** Baseline characteristics of the whole study population and of the subgroup of patients with hs-CRP values.

Variables	All *n* = 500	hs-CRP Group *n* = 400	*p*
Age, years	47 ± 11	47 ± 11	0.998
Gender, M (%)	256 (51)	183 (46)	0.0001
Body mass index, kg/m^2^	27.3 ± 3.6	27.3 ± 3.6	0.997
SBP, mm Hg	149 ± 17	149 ± 17	0.999
DBP, mm Hg	91 + 12	91±12	0.998
Heart rate, bpm	72 ± 9	73 ± 9	0.098
Total cholesterol, mg/dL	205 ± 31	205 ± 31	0.997
Smokers, No (%)	78 (16)	75 (19)	0.246
Fasting glucose, mg/dL	95 ± 11	95 ± 11	0.998
LDL cholesterol, mg/dL	130 ± 31	129 ± 31	0.631
HDL cholesterol, mg/dL	52 ± 12	52 ± 12	0.997
Triglycerides, mg/dL	116 ± 40	116 ± 41	0.999
e-GFR, ml/min/1.73/m^2^	85 ± 20	88 ± 18	0.020
ALT, UI/L	21±11	21 ± 11	0.999
AST, UI/L	22 ± 9	22 ± 9	0.998
γ-GT, UI/L	33 ± 14	32 ± 14	0.287
hs-CRP, mg/L		4.1 ± 2.2	
FBF baseline, mL·0.100 tissue^−1^·min^−1^	3.4 ± 0.7	3.3 ± 0.6	0.024
FBF maximal response to acetylcholine, % of increase	303 ± 180	318 ± 183	0.218
Response to sodium nitroprusside, % of increase	317 ± 110	315 ± 107	0.784
Vascular resistance, U	34 + 8	34 + 7	0.998

ALT = alanine transaminase; AST = aspartate transaminase; SBP = systolic blood pressure; DBP = diastolic blood pressure; FBF = forearm blood flow; γ-GT = gamma glutamyltransferase; LDL= low-density lipoproteins; HDL = high-density lipoproteins; hs-CRP = high-sensitivity C-reactive protein.

**Table 2:** Univariate relationships between FBF maximal response to acetylcholine and different covariates.

	*R*	*p*
γ-GT, UI/L	−0.587	0.0001
ALT, UI/L	−0.559	0.0001
AST, UI/L	−0.464	0.0001
e-GFR, mL/min/1.73/m^2^	0257	0.0001
Gender, male vs. female	0.191	0.0001
Age, years	−0.171	0.0001
BMI, kg/m^2^	−0.152	0.0001
Fasting glucose, mg/dL	−0.101	0.012
Systolic blood pressure, mmHg	−0.055	0.0108
Diastolic blood pressure, mmHg	−0.031	0.248
Smoking, yes vs. no	0.028	0.269
LDL cholesterol, mg/dL	−0.024	0.300
Triglycerides, mg/dL	−0.007	0.435
HDL cholesterol, mg/dL	0.001	0.494

Data are expressed as Pearson product-moment correlation coefficient (*r*) and *p* values. ALT = alanine transaminase; AST = aspartate transaminase; BMI = body mass index; γ-GT = gamma glutamyltransferase; HDL = high-density lipoproteins; LDL = low-density lipoproteins.

**Table 3:** Multiple linear regression models for FBF maximal response to acetylcholine.

	Partial r^2^	Total r^2^	b Coefficient	*P*
γ-GT	35.4	35.4	−0.362	0.0001
ALT	12.3	46.7	−0.297	0.0001
AST	5.4	52.1	−0.217	0.0001
e-GFR	1.9	54.0	0.199	0.0001
Gender	2.1	56.1	0.166	0.0001
Smoking	0.4	56.5	−0.061	0.044

ALT = alanine transaminase; AST = aspartate transaminase; γ-GT = gamma glutamyltransferase.

**Table 4:** Multiple logistic analysis of endothelial dysfunction.

	OR	95% CI	*p*
γ-GT, 10 UI/L	1.927	1.548–2.399	0.0001
ALT, 10 UI/L	2.175	1.608–2.941	0.0001
AST, 10 UI/L	1.973	1.369–2.788	0.0001
Gender, male vs. female	2.695	1.413–5.141	0.003
e-GFR, 10 mL/min/1.73/m^2^	0.699	0.583–0.837	0.0001

ALT = alanine transaminase; AST = aspartate transaminase; γ-GT = gamma glutamyltransferase.

**Table 5:** Multiple logistic analysis of endothelial dysfunction in Subgroup A (*n* = 247).

	OR	95% CI	*p*
γ-GT, 10 UI/L	2.089	1.545–2.823	0.0001
ALT, 10 UI/L	1.556	1.045–2.316	0.029
AST, 10 UI/L	1.865	1.146–3.035	0.019
Gender, male vs. female	2.742	1.147–6.558	0.023
e-GFR, 10 mL/min/1.73/m^2^	0.656	0.522–0.825	0.0001

**Table 6:** Multiple logistic analysis of endothelial dysfunction in Subgroup B (*n* = 253).

	OR	95% CI	*p*
γ-GT, 10 UI/L	1.819	1.335–2.480	0.0001
ALT, 10 UI/L	2.733	1.747–4.273	0.0001
AST, 10 UI/L	1.831	1.141–2.937	0.012
Gender, male vs. female	2.014	0.827–4.904	0.123
e-GFR, 10 mL/min/1.73/m^2^	0.710	0.558–0.903	0.005
